# Supplementary material for: POLRMT mutations impair mitochondrial transcription causing neurological disease
Source: Nat Commun. 2021 Feb 18;12:1135. doi: 10.1038/s41467-021-21279-0 (PMC7893070; doi:10.1038/s41467-021-21279-0)
Supplement: Supplementary file 1 — Supplementary Information [file 41467_2021_21279_MOESM1_ESM.pdf]

SUPPLEMENTARY NOTE 1

***POLRMT* mutations impair mitochondrial transcription causing neurological disease**
Oláhová, Peter *et al*.

Table of Contents

1. **Supplementary results**
   1.1 Case reports
2. **Supplementary materials and methods**
   2.1 Next generation sequencing analysis
   2.2 Thermal stability measurements
   2.3 Mitochondrial network and nucleoid analysis
3. **Supplementary figures**
   3.1 Supplementary Figure 1 Characterisation of the c.2641-1G>C *POLRMT* variant on splicing in P3 and control fibroblasts
   3.2 Supplementary Figure 2 *POLRMT* mutations do not affect thermal stability.
   3.3 Supplementary Figure 3 *In vitro* transcription activity of wild-type and mutant POLRMT
   3.4 Supplementary Figure 4 Analysis of mitochondrial network and nucleoid morphology in *POLRMT* patient fibroblasts
4. **Supplementary tables**
   4.1 Supplementary Table 1 Clinical summary of patients carrying *POLRMT* variants
   4.2 Supplementary Table 2 Primer and template sequences used in this study
5. **References**

1. SUPPLEMENTARY RESULTS

1.1 Case reports

Patient 1

Patient 1 (P1) is a 17-year-old female of mixed ancestry born to healthy, non-consanguineous unrelated parents. Around one year of age, she presented with genu varum secondary to hypophosphatemic rickets and renal Fanconi syndrome. She was also noted to have mild developmental delays, hypotonia and short stature. Brain MRI at the age 5 years demonstrated mild white matter loss with thinning of the corpus callosum. She was treated with phosphate and calcitriol for her rickets with improved skeletal outcome. She attained final height at age 13 years, with height Z score of -4.1. This patient and her laboratory investigations have been described extensively before in the literature (1) (see also Table 1).

Patient 2

Patient 2 (P2) is an 20-year-old Caucasian female. She displayed mild hypotonia and breath holding spells at one month of age and initial examination found bilateral ptosis (left > right). Her gross motor skills were mildly delayed and fine motor and speech skills were found to be behind 1 year. Brain MRI at 8 years was essentially normal and follow up MRI at 17 years of age showed a few scattered nonspecific FLAIR signal hyperintense lesions within the frontoparietal white matter. At age 13 years she was found to have a mild left ear sensorineural hearing loss (SNHL), primarily at higher frequencies. She continues to have mild intellectual disability and a significant impairment in speech and fine motor skills. At age 18 years her neurological examination was normal other than the mild bilateral ptosis, severe left sided SNHL requiring a hearing aid and mild left eye esotropia. She also developed psychiatric symptoms in her teenage years with anxiety and depression and at age 19 years began having psychotic features with severe emotional outbursts and anger and stopped participating in self care.

Patient 3

Patient 3 (P3) is a 58-year-old Caucasian man (weight 72.9 kg, height 1.79 m) who initially presented with a 20-year history of progressive ptosis. Clinical examination revealed bilateral ptosis (<1/3rd of the pupil involved; with evidence of surgical corrective ptosis scars bilaterally) and ophthalmoplegia (restriction of gaze: abduction <60%: adduction: <30%: down gaze 30%; up gaze 10-30%). Proximal myopathy was conspicuously absent. A maternal uncle was reported to have ptosis but this was never clinically-confirmed. Muscle creatine kinase levels were normal at 154 U/L (range 0–220); and lactate levels were normal in blood and CSF (1.1 mmol/l; normal range 0.7–2.1 mmol/l). Brain MRI was normal. Muscle creatine kinase and lactate levels in blood and CSF were normal.

Patient 4

Patient 4 (P4) is a 3-year-old Caucasian boy born to healthy, non-consanguineous parents. Prenatal history was unremarkable. He was born full term at 40 weeks gestational age. Birth weight 3315 g, length 52 cm, and head circumference 36.5 cm. No dysmorphic features on examination. At his last evaluation, he was normocephalic (OFC: 51 cm, 75th centile), height 83 cm (<3rd centile), and weight 12.3 kg (10th centile). He presents with global mild to moderate developmental delay, expressive-receptive language difficulties, short stature, and hypotonia. He was found to have incidentally renal hypomagnesemia (0.6 mg/dl; normal range 1.7–2.4 mg/dl) during workup at age 10 months for febrile urinary tract infection. Magnesium levels have remained low despite supplementation (range 0.6 to 2.1 mg/dl). Phosphorus was

also low at 1.5 mg/dl but responded well to supplementation. Renal ultrasound showed medullary nephrocalcinosis. He has persistent elevation of lactate ranging from 2.4 to 3.8 mmol/l (normal range, 0.7-2.1 mmol/l). He developed one episode of anaemia requiring a blood transfusion attributed to transient erythroblastopenia of childhood. No recurrence of anaemia during follow up. He has generalized hypotonia and stable walk. The patient points to communicate and has a vocabulary of just a few words. He is making vocalizations and using few signs with speech therapy. He seems to understand better simple commands at age 3 years.

Patient 5

Patient 5 (P5) is a 10-year-old Iranian male, the only affected child of healthy consanguineous parents. He was born at term with unremarkable pregnancy and neonatal period with OFC 31 cm, weight 2800 g, and length 48 cm. He has a severe global developmental delay including head control: 13months, sitting: 4 years, walking: 8 years and never developed speech. He has no history of seizure. His vision and hearing are normal. At the age of 10 years, weight was 23 kg (-1.6SD), OFC was 45 cm (-6.0 SD) and height was 112 cm (-4 SD). He had normal facial appearance except of strabismus and large ear. He was unable to walk without assistance. Brain MRI showed ventriculomegaly. Developmental testing at age 10 years by Wechsler Intelligence Scale for Children (HAWIK-IV) showed an IQ 20 in the range of severe to profound intellectual disability.

Patient 6

Patient 6 (P6) is a 14-year-old Estonian female, born to healthy non-consanguineous parents. She has healthy younger brother. She was born from first pregnancy on 37th week of gestation with birth weight 2690g (-1 SD), length 47 cm (-1 SD), OFC 32 cm (-1.5 SD) and Apgar score 10/10. On 32nd week of pregnancy, the mother had threat of premature delivery. After birth, bilateral clubfoot was diagnosed and operated and presented with significant muscular hypotonia and feeding difficulties in infancy. At the age of 8 months she presented with developmental delay, muscular hypotonia and some dysmorphic features - epicanthal folds, strabismus, upturned nose, opened mouth appearance. Her height was 66 cm (-2.5 SD), weight 7 kg (-2 SD), OFC 40 cm (-2.5 SD). Metabolic investigations showed mildly elevated serum lactate 2.4 mmol/L and unspecific organic aciduria (increased excretion of Krebs cycle metabolites and methylmalonate). Brain MRI showed cavum septi pellucidi. At the age of 5 years focal epilepsy was diagnosed. She received antiepileptic treatment for five years, but it was stopped due to normal EEG. Presently she has severe intellectual disability and no speech. She has behavioral problems – irritability and aggressiveness. At the age of 14 years, she presented short stature - her height was 139 cm (-4 SD), weight 37,3 kg (-2 SD) and microcephaly - OFC 49.5 cm (-3.5 SD), BMI=19.3 (50 percentile). Hearing is normal. She has normal signs of puberty. She is not able to dress herself, has partial toileting skills and is able to understand some commands. Periodically low folate level was detected and treated with supplementation. Since 14 years of age additional treatment with coenzyme Q10, riboflavin and L-carnitine was started. This treatment showed some positive effect – better sleep at night and less irritability.

Patient 7

Patient 7 (P7) is a 59-year-old male from a non-consanguineous family of Italian ancestry who has an affected male sibling (Patient 8). His weight was 69.9kg, height 1.72 metres and BMI 23.8. He presented for evaluation in his early 40’s, when he started experiencing difficulty climbing stairs. Since that time, he developed progressive proximal more than distal weakness of his arms and legs, accompanied by muscle atrophy. He recalls normal early development and was very active in sports during childhood. Neurological examination was notable for

sparing of facial and neck muscles, atrophy of the biceps and quadriceps muscles, and diffuse, proximally predominant muscle weakness. There were no elbow or knee contractures, and no calf pseudohypertrophy. Serum creatine kinase levels were elevated 873 U/L (normal 175U/L).

Patient 8

Patient 8 (P8) is the affected 72-year-old male sibling of P7 who has similar clinical and histopathological features. His weight was 65kg, height 1.66 metres and BMI 24.5. He initially presented in his early 40’s when he started experiencing difficulty climbing stairs and rising from the floor. Since then his weakness has been slowly progressive with proximal muscle involvement more prominent than distal involvement. There is no facial involvement, but he has neck flexion and extension weakness. He uses a cane for walking short distances and a wheelchair for longer distances. Neurological examination is notable for right exotropia and a few beats of nystagmus on lateral gaze, no facial weakness, head drop with neck flexion and extension weakness, atrophied biceps muscles, no calf pseudohypertrophy, and diffuse weakness that was more prominent in the proximal muscles. Reflexes were depressed throughout and absent at the biceps. There was no calf pseudohypertrophy. Serum creatine kinase levels were mildly elevated to 307 U/L (normal 175U/L).

2. SUPPLEMENTARY MATERIALS AND METHODS

2.1 Next generation sequencing analysis

Whole exome sequencing (WES), whole genome sequencing (WGS) or nuclear gene panel sequencing, followed by filtering and candidate variant analysis was undertaken in all patients.

For P1 DNA was fragmented by sonication performed on a Covaris S2 system (Covaris, Woburn, MA). Sequencing libraries were constructed using the New England BioLabs NEBNext® Ultra™ DNA Library Prep Kit. Exomes were captured by the SeqCap Kit V3 (NimbleGen) according to the manufacturer's protocol. Libraries were sequenced on an Illumina HiSeq 2500 system at the University Hospital in Motol as described in (2). The resulting FASTQ files were aligned to the human reference genome (hg19) using Novoalign v.2.08.03 (Novocraft Technologies, Selangor, Malaysia). After genome alignment, conversion of SAM format to BAM and duplicate removal were performed with Picard Tools v.1.129. The Genome Analysis Toolkit (GATK v.3.5) was used for local realignment around indels, base recalibration, and variant recalibration and genotyping. Variants were annotated with SnpEff 3.6 using Ensembl gene annotation version.

For P2 DNA was fragmented by sonication performed on a Covaris E220 system (Covaris, Woburn, MA). Sequencing libraries were constructed using the Kapa Hyper Library Prep Kit (Roche). Targeted 406 gene mitochondrial nuclear panel (https://www.newbornscreening.on.ca/sites/default/files/mitochondrial_testing_gene_list_2018_v1.4.pdf) was captured by the SeqCap Kit V3 (NimbleGen) according to the manufacturer's protocol. Libraries were sequenced on an Illumina MiSeq system. The resulting FASTQ files were aligned to the human reference genome (hg19) using BWA-mem options -a -M (v0.7.12-r1039). After genome alignment, conversion of SAM format to BAM and duplicate removal were performed with Picard Tools v.1.134. The Genome HaplotypeCaller (GATK v.3.5) was used for local realignment around indels, base recalibration, and variant recalibration and genotyping. Variant calling is performed per sample by GATK 3.5 at each CCDS exon with a 10 bp padding. Annovar is used to annotate the variant call set. Tertiary analyses are performed at Tute Genomics. Variants are filtered initially with at MAF < 3%.

For P3, WES was performed as previously described (3). Briefly, exome capture was attained using the Illumina TruSeq 62 Mb capture kit, sequenced using the Illumina HiSeq 2000 system in 100 bp reads and aligned to the human reference genome (UCSC hg19). Variants were restricted to those with a minor allele frequency of ≤ 0.01% from in-house controls (*n* = 378) and external variant databases GnomAD (http://gnomad.broadinstitute.org/), National Heart, Lung, and Blood Institute Exome Sequencing Project (http://evs.gs.washington.edu/EVS/) and 1000 Genomes Project (http://www.internationalgenome.org/). On the basis of no confirmed family history, autosomal dominant (heterozygous) and autosomal recessive (homozygous and compound heterozygous) variants were equally considered. First, pathogenic or likely pathogenic variants in known nuclear genes associated with mitochondrial DNA maintenance disorders were excluded including genes that were analysed diagnostically (*POLG, SLC25A4, TWNK, RRM2B, POLG2, TK2* and *RNASEH1*). Next, variants were filtered using Gene Ontology (GO)-Terms to prioritise nuclear genes encoding mitochondrially-targeted proteins and/or DNA repair or replication machinery. GO-Terms employed were the wildcard term 'mitochondr*', DNA repair', 'replication', 'transcription', 'nucleotide', 'purine', 'pyrimidine', 'exonuclease', 'polymerase', 'topoisomerase', 'ligase', 'helicase' and 'nucleoside'. Copy number variants (CNVs) were also examined using the same GO-Terms. *In silico* tools

Polyphen-2 (http://genetics.bwh.harvard.edu/pph2/), Align GVGD (http://agvgd.hci.utah.edu/agvgd_input.php) and SIFT (http://sift.jcvi.org/) were used to assess the pathogenicity of identified candidate variants. The identified *POLRMT* variant in P3 was confirmed by Sanger sequencing of PCR products amplified using custom forward and reverse primers flanked with universal M13-derived tags, using the BigDye Terminator v3.1 Cycle Sequencing kit on the ABI 3130xl Genetic Analyser, according to manufacturer's guidelines. Total RNA was extracted from P3 and control cultured fibroblasts using the ReliaPrep RNA Cell MiniPrep system (Promega). To inhibit nonsense-mediated decay, cultured fibroblast medium was supplemented with 100μg ml^{-1} emetine for 10 hours. RNA was reverse transcribed to cDNA using the GoScript Reverse Transcription system (Promega). Custom forward and reverse primers flanked with universal M13-derived tags were used to amplify *POLRMT* exons 10-13. PCR products were separated by gel electrophoresis and sequenced as described above. Primers available upon request. For completeness, we also performed WGS (30X coverage) using a TruSeq DNA library and the Illumina HiSeq 2000 platform; no additional candidate variants were identified.

For P4, WES performed at GeneDx. Using genomic DNA from the submitted specimen, the Agilent Clinical Research Exome kit was used to target the exonic regions and flanking splice junctions of the genome. These target regions were sequenced simultaneously by massively parallel (NextGen) sequencing on an Illumina HiSeq sequencing system with 100bp or greater paired-end reads. Reads are aligned to human genome build GRCh37/UCSC hg19, and analyzed for sequence variants using a custom-developed analysis tool (Xome Analyzer). Capillary sequencing or another appropriate method is used to confirm all potentially pathogenic variants identified in the individual and relative samples, if submitted. Sequence alterations are reported according to the Human Genome Variation Society (HGVS) nomenclature guidelines. Analysis of exome for the proband includes evaluation of variants that are identified to be de novo, compound heterozygous, homozygous, heterozygous and X-linked in addition to relevant analysis based on the family structure and reported phenotype.

Trio WES analysis was performed on genomic DNA extracted from white blood cells of P5 and parents as previously described in (4). The Agilent SureSelectXT Human All Exon V5 enrichment kit was used to enrich exonic sequences and 100 bp paired-end sequencing was performed on an Illumina HiSeq sequencer. MERAP pipeline was employed for sequence analysis.

Trio WES analysis was performed in the family of P6 using genomic DNA extracted from blood. Sequencing libraries were made using SureSelect Human All Exon V5 kit (Agilent) and target regions were sequenced on Illumina HiSeq platform. The variant analysis was focused on coding nonsynonymous recessive (homozygous and compound heterozygous) and *de novo* variants with allele frequencies below 1% in population databases ExAC and gnomAD. This analysis revealed a *de novo* stop-gain variant c.1525C>T p.Arg509Ter in *SLC22A31*, which was not prioritized due to having 23 heterozygous carriers in gnomAD and for showing a low probability of being loss-of-function intolerant (pLI score = 0 in gnomAD). Recessive variants search revealed putative variants in *OR6A2, KMT2D, TGM7, MMP25, OR7G3*, *POLRMT* and *P2RY11*. While only *KMT2D* is a known disease gene, both detected variants were classified as benign in ClinVar, and the established inheritance pattern (dominant with *de novo* variants) does not fit with known disease association of Kabuki syndrome.

Whole exome sequencing was performed on genomic DNA samples from P7 and P8 using the Illumina platform using previously described methods (5-8). The exome-sequencing pipeline

included sample plating, library preparation using the Illumina Nextera kit (2-plexing of samples per hybridization), hybrid capture using the Illumina Rapid Capture Enrichment kit (37 Mb target), sequencing (150 bp paired reads), sample identification QC check, and data storage. The BWA aligner was used for mapping reads to the human genome build 37 (hg19). Single Nucleotide Polymorphism (SNPs) and insertions/deletions (indels) were jointly called across all samples using Genome Analysis Toolkit (GATK) HaplotypeCaller package version 3.4. Default filters were applied to SNP and indel calls using the GATK Variant Quality Score Recalibration (VQSR) approach. Lastly, the variants were annotated using Variant Effect Predictor (VEP). To select the variants predicted to have high likelihood of pathogenicity, the variant call set was uploaded to the Seqr platform (https://seqr.broadinstitute.org) and filtered using standard online databases as previously described, based on a presumed autosomal recessive inheritance pattern (5-8). Exons 3 and 13 of *POLRMT* were amplified by PCR from genomic DNA samples to confirm specific variants by Sanger sequencing and determine segregation patterns of these variants among affected and unaffected family members.

2.2 Thermal stability measurements

The fluorescent dye Sypro Orange (Invitrogen; λ_{ex} = 490 nm, λ_{em} = 570 nm) was used to monitor the temperature-induced unfolding of POLRMT variants. Proteins were set up in 96 well PCR plates at a final concentration of 1.6 µM protein and 2.5X dye in assay buffer (25 mM Tris-HCl pH 8.0, 0.2 M NaCl, 0.5 mM EDTA, 10 % (w/v) glycerol and 1 mM DTT). Analyses were performed in a C1000 Thermal Cycler using the CFX96 real time software (BioRad). Scans were recorded using the HEX emission filter (560-580 nm) between 4 to 95 °C in 0.5 °C increments with a 5 s equilibration time. Data were normalised between 0-1 and the melting temperature (T_m) was determined from the first derivative of a plot of normalised fluorescence intensity versus temperature (9).

2.3 Mitochondrial network and nucleoid analysis

Control and *POLRMT* patient fibroblasts (9 000 cm^{-2}) were grown in 35 mm µ-Dishes (Ibidi) overnight. For analysis of mitochondrial networks, cells were incubated in 5 nM TMRM (Invitrogen) for 30 minutes. Nucleoids were visualised by incubation in 1.5 $\mu l.ml^{-1}$ PicoGreen (Invitrogen) for 30 minutes prior to staining with 10 nM TMRM. Z-stack images (1024 x 1024, 0.063 µm per pixel) comprising 0.1 µm steps were captured on an inverted array-scanning confocal iSIM microscope (VisiTech) with a TiE body (Nikon) using a 100 X magnification oil immersion objective, and two Hamamatsu Orca Flash 4 sCMOS monochrome cameras. For network analysis using imaging of TMRM alone, a 561 nm laser was used at 85% power for 300 ms. For dual imaging of mitochondrial nucleoids and network, a 488 nm laser (7%) and 561 nm laser (50%) were used to excite PicoGreen and TMRM, respectively, for 400 ms. A 525/50 filter was used for the 488 nm channel, and a 590L filter was used for the 561 nm channel. For quantification of mitochondrial networks, triplicate experiments were undertaken with microscope and laser settings consistent throughout and on each experimental day 10 cells were imaged for each cell line (at least 8 fields). Images were then processed using Richardson-Lucy deconvolution (10 iterations) on NIS-Elements (Nikon). Maximum intensity projections of Z-stacks (opened in Fiji using the Autoscale function) were generated using Fiji then analysed using the MiNA 2.0.0 toolset (10). For pre-processing the following settings were used: blocksize (127), histogram bins (256), maximum slope (3), median filter: radius (2), unsharp mask: radius (3) and mask strength (0.85). Tophat was not applied. For each image,

mean branch length and mean number of branches per mitochondrial network were recorded, and then multiplied to calculate mean mitochondrial network length per image.

3. SUPPLEMENTARY FIGURES

3.1 Supplementary Figure 1

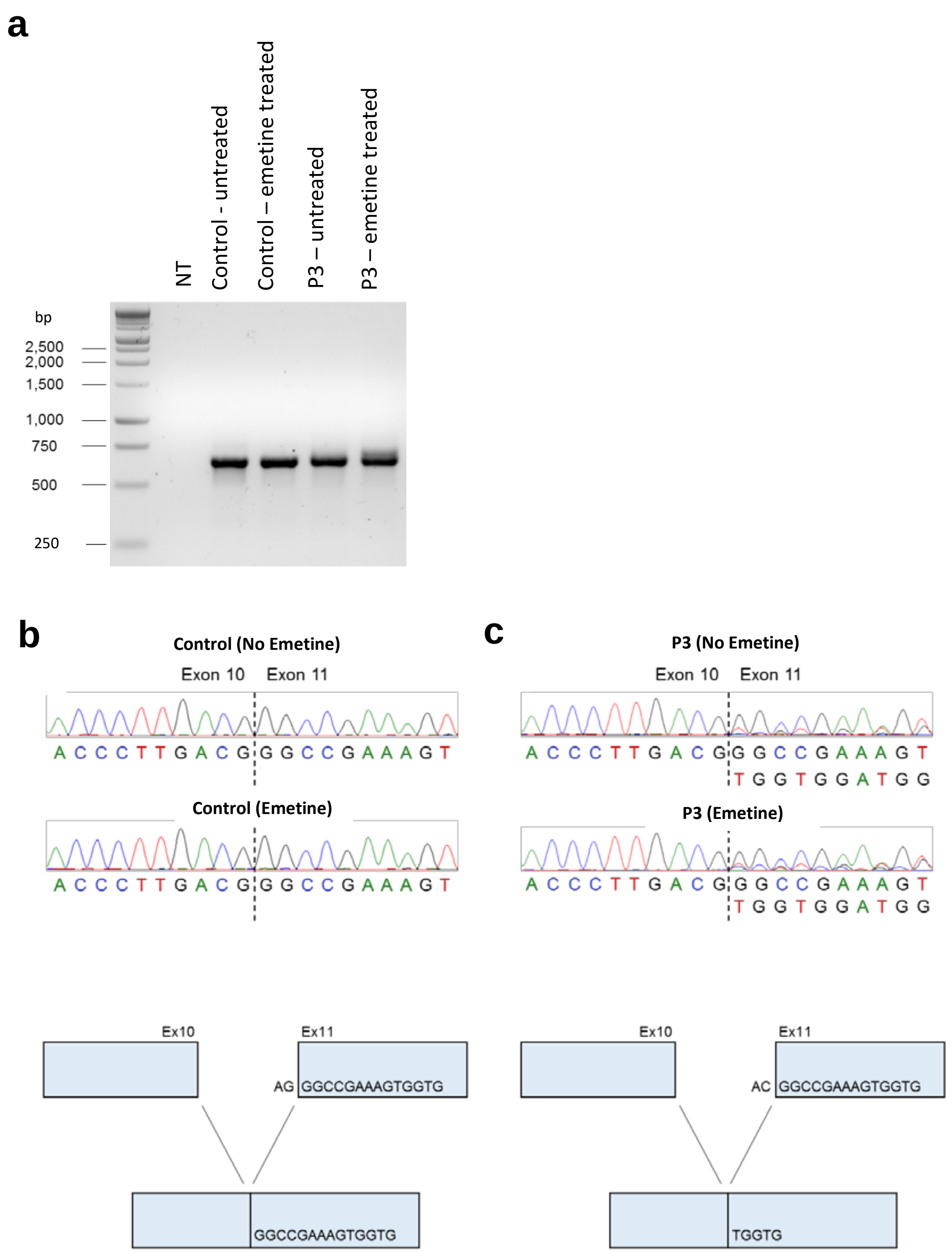

Supplementary Figure 1 Characterisation of the c.2641-1G>C *POLRMT* variant on splicing in P3 and control fibroblasts. (**A**) Amplification of control and P3 fibroblast-derived cDNA across *POLRMT* exons 10-13 and sequencing chromatograms showing PCR products from (**B**) control and (**C**) P3 fibroblasts without and with emetine treatment (n = 2). Nonsense-mediated decay was inhibited in control and P3 fibroblasts with 100µg ml^{-1} emetine for 10 hours. NT – no template, bp – base pairs.

3.2 Supplementary Figure 2

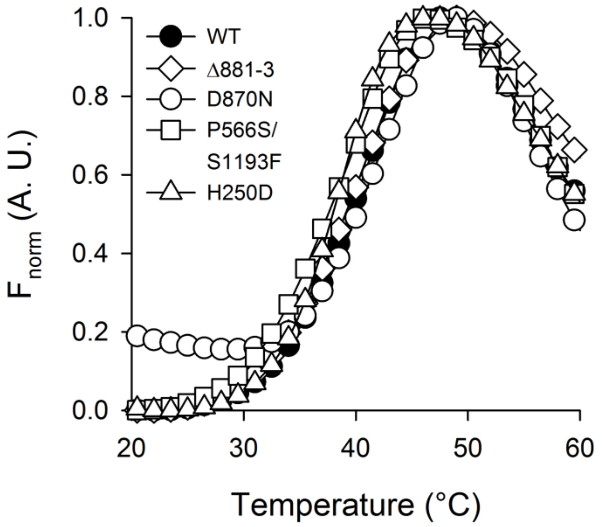

Variant	T_m (C)
WT	41.4
D870N	41.5
P566S/ S1193F	40.3
H250D	39.3
Δ881-3	41.8

Supplementary Figure 2 *POLRMT* mutations do not affect thermal stability. The *POLRMT* variants corresponding to P1 (D870N, P566S/S1193F), P2 (H250D) and P3 (del881-3) were analysed. The del742-7 variant from P2 could not be expressed in sufficiently high quantities for analysis. Differential scanning fluorimetry measurements of wild-type and mutant POLRMT showed no significant effect on stability due to the mutations. The melting temperature (T_m) for each variant was determined from the first derivative of a plot of normalised fluorescence intensity versus temperature (9).

3.3 Supplementary Figure 3

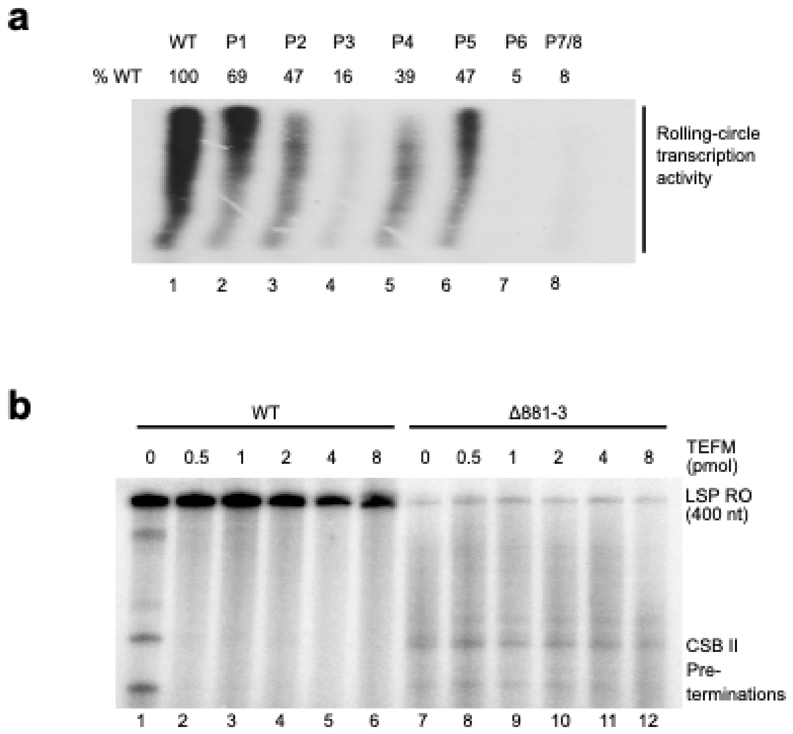

Supplementary Figure 3 *In vitro* transcription activity of wild-type and mutant POLRMT. (**A**) Promoter-dependent transcription from a circular LSP template using the combination of mutations identified in affected patients. Transcription products of defined length are not formed. (**B**) *In vitro* transcription assays were performed on a linear LSP template with increasing amounts of the elongation factor TEFM. Both wild-type and Δ881-3 POLRMT show pre-termination products and stalling at CSB II in the absence of TEFM (lanes 1 and 7, respectively). Addition of TEFM results in the removal of these stalled products in wild-type POLRMT (lanes 2-6) but not in the Δ881-3 variant (lanes 8-12).

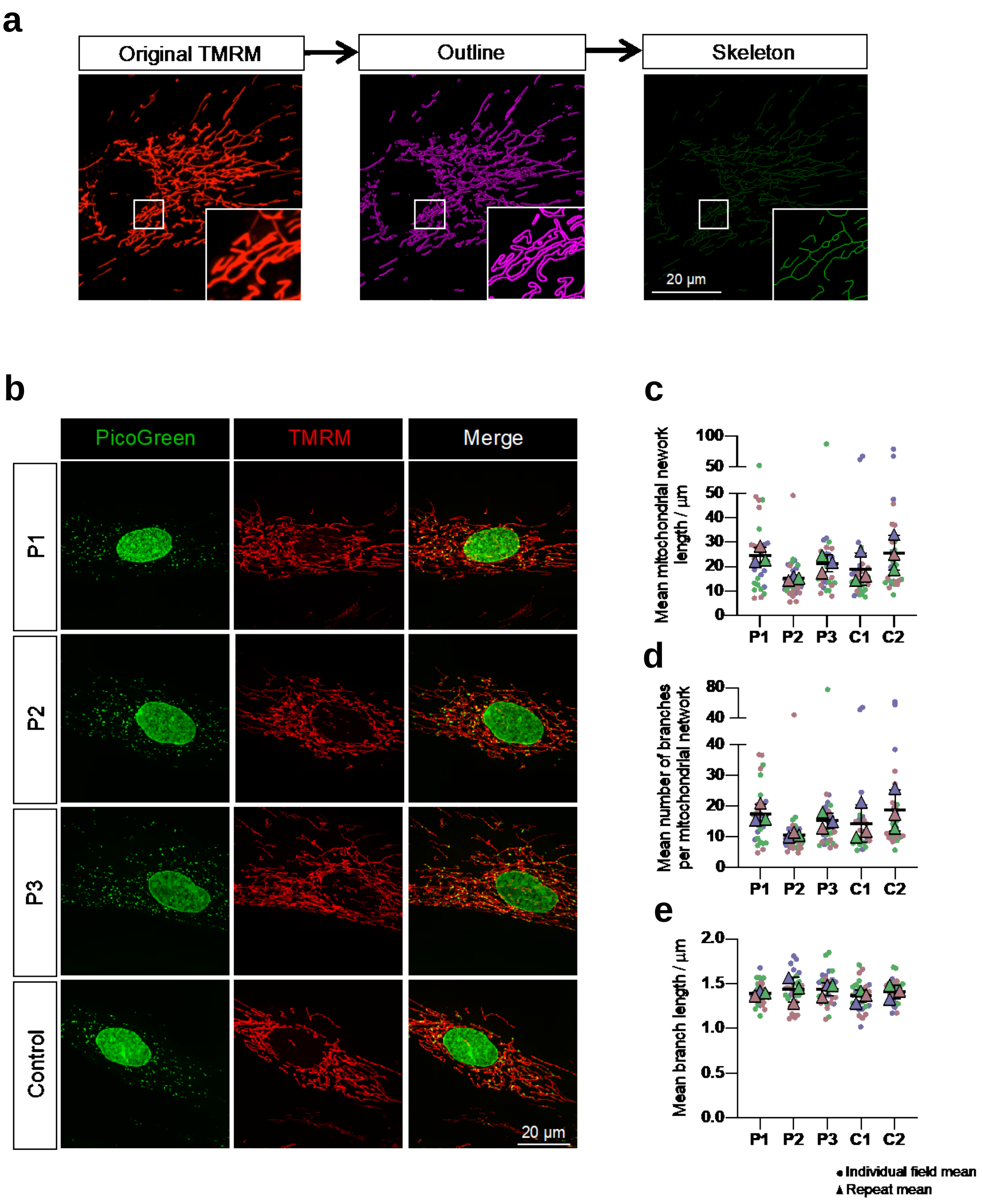

Supplementary Figure 4 Analysis of mitochondrial network and nucleoid morphology in *POLRMT* patient fibroblasts. (**A**) The MiNA toolset was used on Fiji to generate a skeleton which was quantified to describe mitochondrial network morphology. (**B**) Representative images of PicoGreen (green) and TMRM-stained (red) *POLRMT* patient and control fibroblasts show normal mitochondrial network and nucleoid distribution. (**C**) No marked difference was observed in the mean mitochondrial network length per cell between control and patient fibroblasts harbouring *POLRMT* variants, nor was any difference identified in (**D**) mean

number of branches per network or (**E**) mean branch length. Circles represent data from individual fields (typically one cell) from three independent repeats, with mean values for each repeat represented by triangles. Error bars +/- SD. Different colours represent three independent experimental days. Statistical analysis *via* one-way ANOVA (Kruskal-Wallis) and Dunn's multiple comparison test was used and demonstrated no significant differences between any cell lines.

4. SUPPLEMENTARY TABLES

4.1 Supplementary Table 1

Clinical summary of patients carrying *POLRMT* variants.

CLINICAL FEATURES	P1	P2	P3	P4	P5	P6	P7	P8
Skeletal abnormality	+	-	-	-	-	+	-	-
Short stature	+	-	-	+	+	+	-	-
Dysmorphic features	-	-	-	-	+	+	-	-
Sensorineural hearing loss	-	+	-	-	-	-	-	-
Vision problems (e.g.Exotropia)	-	-	-	-	+	+	-	+
Developmental delay	+	+	-	+	+	+	-	-
Hypotonia	+	-	-	+	+	+	+	+
Incontinence (bladder/bowels)	+	+	-	-	-	-	-	-
Epilepsy	-	-	-	-	-	+	-	-
Ptosis	-	+	+	-	-	-	-	-
Eye movement abnormality (e.g. Nystagmus/PEO)	-	+	+	-	-	+	-	+
Facial muscle weakness/wasting	-	-	+	-	-	+	-	-
Neck ptosis-weakness/wasting	-	-	-	-	-	-	-	+
Limb Girdle involvement (Myopathy)	-	-	-	-	+	-	+	+
Neuropathy	-	-	-	-	-	n.d.	-	+
Contractures	-	-	-	-	-	+	-	-
Gastrointestinal involvement	-	+	-	+	-	+	-	-
Renal abnormalities	+	-	-	+	-	-	-	-
Respiratory problems	-	-	-	-	-	+	-	+
Electrolyte abnormalities	+	-	-	+	n.d.	-	-	-
Anaemia	-	-	-	+	n.d.	+	-	-
Endocrine dysfunction	+	-	-	-	n.d.	+	-	-
MRI brain abnormalities	+	+	-	-	+	+	-	-

n.d. = not determined

4.2 Supplementary Table 2

Primer and template sequences used in this study

Primer/Template	Sequence (5'-3')	Comments
H250D	FW: GCTGGTCGTCCACGACGGCCAGCGGCAG	**Site-directed mutagenesis**
	RV: CTGCCGCTGGCCGTCGTGGACGACCAGC	
P566S	FW: CCTGCGGGAGCAGTCCTGGCCCCTGCCAG	
	RV: CTGGCAGGGGCCAGGACTGCTCCCGCAGG	
D870N	FW: CGGAGGAGGTGATGGATAACATCCTGGACTCCGCGGAC	
	RV: GTCCGCGGAGTCCAGGATGTTATCCATCACCTCCTCCG	
S1193F	FW: CCTGGTCAAGCGGTTCTGCTTTGAGCCCCAGAAGATCTTG	
	RV: CAAGATCTTCTGGGGCTCAAAGCAGAACCGCTTGACCAGG	
Δ881-3	FW: ACCAACCCTTGACGTGGTGGATGGGCGC	
	RV: GCGCCCATCCACCACGTCAAGGGTTGGT	
Δ742-7	FW: CCCTCCGAGGCGCCCCAGCCGCACAGCGCCGCG	
	RV: GGCGGGCGCGGCGCTGTGCGGCTGGGGCGCCTC	
S611F	FW: CCACGTGTATTTCTTCCGCAACG	
	RV: CGTTGCGGAAGAAATACACGTGG	
F641L	FW: CCACGCTGACCTTGGAGGCGGTGG	
	RV: CCACCGCCTCCAAGGTCAGCGTGG	
P810S	FW: CCTACCCCTGCTCGCCGCACTTCAACC	
	RV: GGTTGAAGTGCGGCGAGCAGGGGTAGG	
R1013C	FW: GCAGATTGAGAAGTGCCTCCGGGAGCTG	
	RV: CAGCTCCCGGAGGCACTTCTCAATCTGC	
SNRI Template	28 -mer template DNA: CGTCTGGCGTGCGCGCCGCTACCCCATG	**SNRI assay**
	28-mer non-template DNA: CATGGGGTAATTATTATTTCGCCAGACG	
	14-mer RNA: AGUCUGCGGCGCGC	
LSP Template	See Falkenberg *et al*. (2002)	***In vitro* transcription**
OriL ssDNA	GGGCTTCTCCCGCCTTTTTT CCGGCGGCGGGAGAAGTAGAT TGAAG	**Primase assay**
D-loop	FW: CTCACCCACTAGGATACCAAC	**Southern blot probes for H-strand mtDNA synthesis**
	RV: GATACTGCGACATAGGGTGC	
28S DNA	FW: GCCTAGCAGCCGACTTAGAACTGG	
	RV: GGGCCTCCCACTTATTCTACACCTC	
ATP6	FW: GTGCATGAGTAGGTGGCCTGC	**qPCR probes**
	RV: TAAGATTAAAAATGCCCTAGCCCACTTC	
CO1	FW: ACGTTGTAGCCCACTTCCAC	

	RV: CATCGGGGTAGTCCGAGTAA	
CYTB	FW: CCTAGGGGGTTGTTTGATCCC	
	RV: GAGGCTACTCAGTAGACAGTCC	
ND1	FW: TAGAGTTCAGGGGAGAGTGCG	
	RV: GCTTCAACATCGAATACGCCGC	
ND6	FW: AGGTAGGATTGGTGCTGTGG	
	RV: CCAATAGGATCCTCCCGAATC	
RNR1	FW: CTAGCCACACCCCCACGGGA	
	RV: CGCGGTGGCTGGCACGAAAT	
mtDNA	FW: AAGGTAGCGGATGATTCAGCC	
	RV: GCCTGCCTGATCCTCCAAAT	
Nuclear DNA	FW: GTGCTCGCGCTACTCTCTCT	
	RV: GATTAACTTGAGAAGGAAGTCACG	

5. REFERENCES

1. Bowden SA, Patel HP, Beebe A, and McBride KL. Successful Medical Therapy for Hypophosphatemic Rickets due to Mitochondrial Complex I Deficiency Induced de Toni-Debre-Fanconi Syndrome. *Case Rep Pediatr.* 2013;2013:354314.
2. Hartmannova H, Piherova L, Tauchmannova K, Kidd K, Acott PD, Crocker JF, et al. Acadian variant of Fanconi syndrome is caused by mitochondrial respiratory chain complex I deficiency due to a non-coding mutation in complex I assembly factor NDUFAF6. *Hum Mol Genet.* 2016;25(18):4062-79.
3. Sommerville EW, Alston CL, Pyle A, He L, Falkous G, Naismith K, et al. De novo CTBP1 variant is associated with decreased mitochondrial respiratory chain activities. *Neurol Genet.* 2017;3(5):e187.
4. Kahrizi K, Hu H, Hosseini M, Kalscheuer VM, Fattahi Z, Beheshtian M, et al. Effect of inbreeding on intellectual disability revisited by trio sequencing. *Clin Genet.* 2019;95(1):151-9.
5. Ghaoui R, Cooper ST, Lek M, Jones K, Corbett A, Reddel SW, et al. Use of Whole-Exome Sequencing for Diagnosis of Limb-Girdle Muscular Dystrophy: Outcomes and Lessons Learned. *JAMA Neurol.* 2015;72(12):1424-32.
6. Reddy HM, Cho KA, Lek M, Estrella E, Valkanas E, Jones MD, et al. The sensitivity of exome sequencing in identifying pathogenic mutations for LGMD in the United States. *J Hum Genet.* 2017;62(2):243-52.
7. Reddy HM, Hamed SA, Lek M, Mitsuhashi S, Estrella E, Jones MD, et al. Homozygous nonsense mutation in SGCA is a common cause of limb-girdle muscular dystrophy in Assiut, Egypt. *Muscle Nerve.* 2016;54(4):690-5.
8. Lek M, Karczewski KJ, Minikel EV, Samocha KE, Banks E, Fennell T, et al. Analysis of protein-coding genetic variation in 60,706 humans. *Nature.* 2016;536(7616):285-91.
9. Matulis D, Kranz JK, Salemme FR, and Todd MJ. Thermodynamic stability of carbonic anhydrase: measurements of binding affinity and stoichiometry using ThermoFluor. *Biochemistry.* 2005;44(13):5258-66.
10. Valente AJ, Maddalena LA, Robb EL, Moradi F, and Stuart JA. A simple ImageJ macro tool for analyzing mitochondrial network morphology in mammalian cell culture. *Acta Histochem.* 2017;119(3):315-26.
