# Supplementary material for: POLRMT mutations impair mitochondrial transcription causing neurological disease
Source: Nat Commun. 2021 Feb 18;12:1135. doi: 10.1038/s41467-021-21279-0 (PMC7893070; doi:10.1038/s41467-021-21279-0)
Supplement: Supplementary file 2 — Description of Additional Supplementary Files [file 41467_2021_21279_MOESM2_ESM.pdf]

Description of Additional Supplementary Files

File Name: Supplementary Movie 1.

Description: Conformational dynamics of Pro566 and Ser1193 during processive transcription. A morph between LSP-bound POLRMT in the initiation state (PDB ID: 6ERQ) and bound to TEFM in the elongation state (PDB ID: 4BOC) was made in Chimera. The Pro566 and Ser1193 residues are shown in magenta. Large conformational changes are seen upon DNA and TEFM binding, although the regions immediately surrounding Pro566 and Ser1193 undergo relatively little movement. The P566S/S1193F mutations may result in increased flexibility in these regions, mimicking the TEFM-induced conformational change and allowing for some processive transcription in the absence of TEFM.
