# Supplementary material for: POLRMT mutations impair mitochondrial transcription causing neurological disease
Source: Nat Commun. 2021 Feb 18;12:1135. doi: 10.1038/s41467-021-21279-0 (PMC7893070; doi:10.1038/s41467-021-21279-0)
Supplement: Supplementary file 5 — Reporting Summary [file 41467_2021_21279_MOESM5_ESM.pdf]

nature research

Corresponding author(s): TAYLOR

Last updated by author(s): Nov 25, 2020

Reporting Summary

Nature Research wishes to improve the reproducibility of the work that we publish. This form provides structure for consistency and transparency in reporting. For further information on Nature Research policies, see our Editorial Policies and the Editorial Policy Checklist.

Statistics

For all statistical analyses, confirm that the following items are present in the figure legend, table legend, main text, or Methods section.

n/a	Confirmed	
☐	☒	The exact sample size (*n*) for each experimental group/condition, given as a discrete number and unit of measurement
☐	☒	A statement on whether measurements were taken from distinct samples or whether the same sample was measured repeatedly
☐	☒	The statistical test(s) used AND whether they are one- or two-sided<br>*Only common tests should be described solely by name; describe more complex techniques in the Methods section.*
☒	☐	A description of all covariates tested
☒	☐	A description of any assumptions or corrections, such as tests of normality and adjustment for multiple comparisons
☐	☒	A full description of the statistical parameters including central tendency (e.g. means) or other basic estimates (e.g. regression coefficient) AND variation (e.g. standard deviation) or associated estimates of uncertainty (e.g. confidence intervals)
☒	☐	For null hypothesis testing, the test statistic (e.g. *F*, *t*, *r*) with confidence intervals, effect sizes, degrees of freedom and *P* value noted<br>*Give P values as exact values whenever suitable.*
☒	☐	For Bayesian analysis, information on the choice of priors and Markov chain Monte Carlo settings
☒	☐	For hierarchical and complex designs, identification of the appropriate level for tests and full reporting of outcomes
☒	☐	Estimates of effect sizes (e.g. Cohen's *d*, Pearson's *r*), indicating how they were calculated

Our web collection on statistics for biologists contains articles on many of the points above.

Software and code

Policy information about availability of computer code

Data collection

Data collection/analysis was performed using the following links:

PDB (https://www.rcsb.org) - The structures of the POLRMT initiation complex (PDB ID: 6ERQ), elongation complex (PDB ID: 4BOC) and POLRMT alone (PDB ID: 3SPA).

For molecular genetic/in silico analysis the following sources were used:

NCBI (https://www.ncbi.nlm.nih.gov/nuccore/) - NM_005035.3
NCBI (https://www.ncbi.nlm.nih.gov/protein/110618253) - NP_005026.3
PolyPhen2 (http://genetics.bwh.harvard.edu/pph2/)
SIFT (https://sift.bii.a-star.edu.sg)
Mutation Taster (http://www.mutationtaster.org)
1000 Genomes Consortium (http://www.internationalgenome.org/home)
Iranome Consortium (http://www.iranome.ir/)
gnomAD (https://gnomad.broadinstitute.org/)
CADD (https://cadd.gs.washington.edu/score)

Data analysis

In silico analysis was performed in PyMol V 1.3 (Schrodinger, LLC) and UCSF Chimera V 1.10.2
he BioRad CFX96 real time software was used to quantify transcript levels
The BioRad imaging system and Image Lab v1.2 was used for immunoblotting analysis
Southern blot analysis and the in vitro transcription assays were quantified using ImageJ software, version 1.53 (PMID: 22930834)

For manuscripts utilizing custom algorithms or software that are central to the research but not yet described in published literature, software must be made available to editors and reviewers. We strongly encourage code deposition in a community repository (e.g. GitHub). See the Nature Research guidelines for submitting code & software for further information.

Data

Policy information about availability of data

All manuscripts must include a data availability statement. This statement should provide the following information, where applicable:

- Accession codes, unique identifiers, or web links for publicly available datasets
- A list of figures that have associated raw data
- A description of any restrictions on data availability

The data that support the findings of this study are available from the corresponding authors upon request.
We believe we have used all the databases/accession codes appropriately and updated the `Data avalibility' section:

*The authors declare that the data supporting the findings of this study are available within the paper and its supplementary information files. Source data are provided with this paper. Accession number for POLRMT cDNA used in the study is NM_005035.3 (https://www.ncbi.nlm.nih.gov/nuccore/NM_005035.3). The structures of the POLRMT initiation complex (PDB ID: 6ERQ), elongation complex (PDB ID: 4BOC) and POLRMT alone (PDB ID: 3SPA) were sourced from PDB (https://www.rcsb.org).

Field-specific reporting

Please select the one below that is the best fit for your research. If you are not sure, read the appropriate sections before making your selection.

☒ Life sciences ☐ Behavioural & social sciences ☐ Ecological, evolutionary & environmental sciences

For a reference copy of the document with all sections, see nature.com/documents/nr-reporting-summary-flat.pdf

Life sciences study design

All studies must disclose on these points even when the disclosure is negative.

Sample size

No sample size calculation was performed because each 'sample' corresponded to an individual patient or control cell line and mutation present in that specific patient e.g. only one skin biopsy was available from each patient and control. The experiments were then performed on duplicates or triplicates from independent biological repeats as indicated in figure legends.

Data exclusions

No data were excluded from the analysis

Replication

As stated in the methods and figure legends the experiments were performed in duplicates or triplicates from independent biological repeats. Where applicable error bars +/- SEM were calculated.

Randomization

The allocation of samples was not random, because specific patient samples or recombinant proteins representing the patient mutations identified in the study were used. We believe that the Editorial question about this has now been addressed.

Blinding

The investigators were not blinded to group allocations during sample collection or data analysis, because specific patient samples or recombinant proteins representing the patient mutations identified in the study were used.

Reporting for specific materials, systems and methods

We require information from authors about some types of materials, experimental systems and methods used in many studies. Here, indicate whether each material, system or method listed is relevant to your study. If you are not sure if a list item applies to your research, read the appropriate section before selecting a response.

Materials & experimental systems

n/a	Involved in the study
	☒ Antibodies
	☒ Eukaryotic cell lines
☒	Palaeontology and archaeology
☒	Animals and other organisms
	☒ Human research participants
☒	Clinical data
☒	Dual use research of concern

Methods

n/a	Involved in the study
☒	ChIP-seq
☒	Flow cytometry
☒	MRI-based neuroimaging

Antibodies

Antibodies used	The following primary antibodies were used in this study: MS601 (ab110411, Abcam), NDUFB8 (ab110242, Abcam), SDHA (ab14715, Abcam), UQCRC2 (ab14745, Abcam), COXI (ab14705, Abcam), ATP5A (ab14748, Abcam) and β-actin (Cloud Clone Corp. CAB340Hu22). HRP-conjugated secondary antibodies: DAKO, P0399, and P0260.<br><br>This information has now been added to the manuscript.
Validation	MS601 (ab110411, Abcam) – according to manufacturer's guidelines 1:1000 dilution was used and a PVDF transfer. Secondary HRP-conjugated anti-mouse antibody was used in 1:2000 dilution. Manufactures' website shows a western blots analysis of the antibody in lysates isolated from Human B cell lymphoma and MRC5 fibroblasts. The antibody was cited in 242 studies (e.g. Mingirulli N et al. Clinical presentation and proteomic signature of patients with TANGO2 mutations. J Inherit Metab Dis N/A:N/A (2019)).<br>NDUFB8 (ab110242, Abcam) - according to manufacturer's guidelines 1:1000 dilution was used. Secondary HRP-conjugated anti-mouse antibody was used in 1:2000 dilution. Manufactures' website shows a western blots analysis of the antibody in lysates isolated from human, cow, rat and mouse heart. The antibody was cited in 111 studies (e.g. Alston et al. Pathogenic Bi-allelic Mutations in NDUFAF8 Cause Leigh Syndrome with an Isolated Complex I Deficiency, AJHG, 106 (1) 92-101 (2020); Sommerville EW et al. Instability of the mitochondrial alanyl-tRNA synthetase underlies fatal infantile-onset cardiomyopathy. Hum Mol Genet 28:258-268 (2019)).<br>SDHA (ab14715, Abcam) - 1:2000 dilution was used. Secondary HRP-conjugated anti-mouse antibody was used in 1:2000 dilution. Manufactures' website shows a western blots analysis of the antibody in lysates isolated from wild type HEK93, SDHA KO HEK293, MCF7 and HepG2, in addition to human, bovine, rat and mouse heart mitochondria. The antibody was cited in 235 studies (Alston et al. A recessive homozygous p.Asp92Gly SDHD mutation causes prenatal cardiomyopathy and a severe mitochondrial complex II deficiency, Human Genetics 134, 869-879 (2015); Thompson K et al. OXA1L mutations cause mitochondrial encephalopathy and a combined oxidative phosphorylation defect. EMBO Mol Med 10:N/A (2018)).<br>UQCRC2 (ab14745, Abcam) - according to manufacturer's guidelines 1:1000 dilution was used. Secondary HRP-conjugated anti-mouse antibody was used in 1:2000 dilution. Manufactures' website shows a western blots analysis of the antibody in lysates isolated from H23, Ramos cell lines and human skeletal muscle. The antibody was cited in 147 studies (Oláhová et al. Molecular genetic investigations identify new clinical phenotypes associated with BCS1L-related mitochondrial disease, Human Molecular Genetics, Volume 28, Issue 22, (2019)).<br>COXI (ab14705, Abcam) - 1:1000 dilution was used. Secondary HRP-conjugated anti-mouse antibody was used in 1:2000 dilution. Manufactures' website shows a western blots analysis of the antibody in lysates isolated from human, bovine, rat and mouse heart. The antibody was cited in 280 studies (e.g. Olahova et al. A truncating PET100 variant causing fatal infantile lactic acidosis and isolated cytochrome c oxidase deficiency, EJHG, 23, 935-939 (2015)).<br>ATP5A (ab14748, Abcam) - 1:1000 dilution was used. Secondary HRP-conjugated anti-mouse antibody was used in 1:2000 dilution. Manufactures' website shows a western blots analysis of the antibody in lysates isolated from human, bovine, rat, mouse heart mitochondria; human liver tissue, HepG2 cells. The antibody was cited in 243 studies (e.g. Olahova et al. Biallelic Mutations in ATP5F1D, Which Encodes a Subunit of ATP Synthase, Cause a Metabolic Disorder, AJHG, 102 (3) 494-504; (2018)).<br>β-actin (Cloud Clone Corp. CAB340Hu22) - The antibody is a mouse monoclonal antibody raised against ACTB. It has been selected for its ability to recognize ACTB in immunohistochemical staining and western blotting. For Western blotting: 1/10000 (1mg/ml). The manufactures' website shows 3 citation for this antibody (e.g. Thompson et al. Targeted Elimination of Senescent Beta Cells Prevents Type 1 Diabetes, Cell Metabolism, 29 (5), 1045-1060, (2019)).

Eukaryotic cell lines

Policy information about cell lines

Cell line source(s)	Fibroblasts-derived cell lines were established from patient skin biopsies. Spodoptera frugiperda (Sf9) insect cells were purchased from Thermo Fisher Scientific. Sf9 cells were used for expression of recombinant proteins.
Authentication	Genomic DNA from the patient cell lines used was sequenced to confirm the presence of pathogenic POLRMT variants. Sf9 cell line used was not authenticated.
Mycoplasma contamination	We routinely confirm all patient-derived cell lines and Sf9 cell lines test negative for mycoplasma contamination
Commonly misidentified lines<br>(See ICLAC register)	No commonly misidentified cell lines were used in the study.

Human research participants

Policy information about studies involving human research participants

Population characteristics	The cohort consists of 8 patients from 7 families. All the patients are carrying distinct pathogenic variants in the POLRMT gene. The age and gender of the pateints was: 16y F, 18y F, 57y M, 3y M, 10y M, 14y F, 59y M, 72y M. One adult patient presented with an adult-onset PEO and two adult patients from the same family only manifested muscle weakness. However, the predominant clinical phenotype of the five paediatric patients is an early onset moderate to severe developmental delay, mild to severe intellectual disability, hypotonia/muscle weakness, short stature, and speech delay. All the clinical presentations are associated with mitochondrial disorders.
Recruitment	Participants were recruited and diagnosed with suspected mitochondrial disease through accredited clinical diagnostic pathways. The online tool, GeneMatcher, facilitated collaboration between the research centres involved (https://genematcher.org).
Ethics oversight	All procedures were in accordance with the ethical principles of the Declaration of Helsinki. Written patient consent was obtained, and all the studies were performed in agreement with the approved guidelines of local ethics committees of each institution that participated in this study.<br><br>We have now provided this information in the manuscript and in the reporting summary:<br><br>"All procedures were in accordance with the ethical principles of the Declaration of Helsinki. Written patient consent was obtained, and all the studies were performed in agreement with the approved guidelines of local ethics committees of each institution that participated in this study with samples stored in the Newcastle Mitochondrial Research Biobank (NRES Committee North East - Newcastle & North Tyneside 1; 16/NE/0267)."<br><br>The authors affirm that human research participants and parents/guardians of involved patients have seen the material to be published and have provided informed consent for publication of the images in Figure 1.

Note that full information on the approval of the study protocol must also be provided in the manuscript.
